# The relationship between achievement goals and scholastic achievement in dental students: the mediating effect of learning engagement

**DOI:** 10.3389/fpsyg.2026.1740774

**Published:** 2026-10-08

**Authors:** Kaiqi Peng, Yonglin Feng, Shanshan Liu, Wengian Lu, Huancai Lin, Yan Zhou

**Affiliations:** 1 Hospital of Stomatology, Guanghua School of Stomatology, Sun Yat-sen University, Guangzhou, China; 2 Department of Psychology, Sun Yat-sen University, Guangzhou, China; 3 Guangzhou Tianhe Middle School, Guangzhou, China; 4 Department of Stomatology, The First Affiliated Hospital of Bengbu Medical University, Bengbu, China

**Keywords:** achievement goals, dental students, learning engagement, mediating effect, scholastic achievement

## Abstract

**Background:**

Scholastic achievement during college is important for the professional careers of dental students. Previous studies have reported that learning engagement may be the mediator between achievement goals and scholastic achievement. This study aims to examine whether learning engagement mediated the relationship between achievement goals and scholastic achievement.

**Methods:**

This study included dental students from two universities in China. This was a quantitative, cross-sectional study. The students were invited to complete an online questionnaire. A total of 679 students participated in the study. The questionnaire consisted of four main parts: demographic characteristics, an achievement goals scale, a learning engagement scale, and a scholastic achievement scale. We conducted a one-way analysis of variance (ANOVA) and correlation analysis using Statistical Package for the Social Sciences (SPSS) 26.0 software. Mplus 8.3 was used to examine the potential mediating effect of learning engagement in a structural equation model.

**Results:**

Findings demonstrated that the associations between achievement goals and the scholastic achievement of dental students were as follows: performance-approach goals (*β* = 0.15, *p* < 0.05), mastery-approach goals (*β* = 0.73, *p* < 0.001), performance-avoidance goals (*β* = −0.03, *p* > 0.05), and mastery-avoidance goals (*β* = −0.10, *p* < 0.05). Learning engagement played a mediating role in the relationship between the three achievement goals (mastery-approach, performance-avoidance, and mastery-avoidance goals) and scholastic achievement. Learning engagement did not mediate the relationship between performance-approach goals and scholastic achievement.

**Conclusion:**

Learning engagement mediated the relationship between achievement goals and scholastic achievement. These findings suggest that dental educators should prioritize cultivating achievement goals among students and enhancing learning engagement to improve professional outcomes.

## 1 Introduction

Medicine is a professional science and art concerned with the maintenance of health and the prevention, alleviation, or cure of disease. In mainland China, most dental students have not acquired medical knowledge before entering medical school. It is not easy to achieve excellent scholastic achievement. The scholastic achievement of medical students can predict their future professional competence in their medical career (Tartas et al., 2011). Poor academic performance in medical school may negatively affect a student’s future medical career. Follow-up studies have demonstrated that students who perform poorly in school face much higher risks later in their careers, and as independent doctors, they are more likely to face disciplinary action by medical boards, make clinical errors, or fail to meet standards of medical care (Papadakis et al., 2005; Papadakis et al., 2008). Previous research has linked motivation to scholastic achievement (Mascareñas and Diola, 2025; Vu et al., 2021). However, the specific psychological mechanisms—particularly how internal drive translates into learning engagement—remain insufficiently explored in the dental education context (Hernández González and Blackford, 2022; Mai et al., 2023). Understanding these factors is not only an academic exercise but also a practical necessity for improving dental education. Therefore, it’s important to identify what is associated with better scholastic achievement and how to improve the scholastic achievement of dental students.

Many scholars have explored this problem from a psychological perspective and found that motivation is associated with scholastic achievement (Edgar et al., 2019; Isik et al., 2017; Sarkis et al., 2020). Motivation drives students to listen carefully, practice skills, complete homework, and ultimately achieve good scholastic achievement. Achievement goals theory is well established as a framework for explaining students’ motivation (Cook and Artino, 2016; Elliot and McGregor, 1999; Weiner, 1990). In achievement goals theory, motivation is interpreted as the general purpose for which students engage in learning, rather than a specific goal. Elliott et al. differentiated achievement goals on two fundamental dimensions: definition (performance or mastery) and valence (approach or avoidance). Therefore, there are four types of achievement goals: performance-approach (P-AP) goals, performance-avoidance (P-AV) goals, mastery-approach (M-AP) goals, and mastery-avoidance (M-AV) goals (Elliot and McGregor, 2001; Scherrer et al., 2024). Performance-approach (P-AP) goals mean that individuals tend to demonstrate competence relative to others, such as aiming to perform well compared with other students. Performance-avoidance (P-AV) goals mean that individuals tend to avoid being perceived as incompetent by others, such as aiming not to perform worse than other students. Mastery-approach (M-AP) goals mean that individuals focus more on improving their self-focused or task-based abilities, such as aiming to master all the material mentioned in class. In addition, individuals with mastery-avoidance (M-AV) goals focus on avoiding self-focused or task-based incompetence, such as aiming to avoid being less than they are capable of. Previous studies have shown that different types of achievement goals have varying associations with scholastic achievement. Studies have reported that performance-approach goals are more strongly associated with score-related outcomes, mastery-approach goals are more strongly associated with deep negative learning, and avoidance goals are more associated with negative outcomes (Cook and Artino, 2016; Hall et al., 2015; Li et al., 2021). Mastery goals focus on competence development, whereas performance goals focus on competence demonstration (Senko et al., 2011). Approach goals direct behavior toward positive outcomes, whereas avoidance goals lead individuals away from negative outcomes (Elliot, 2005). Because dental education involves both clinical skill acquisition and competitive academic assessments, each goal orientation involves distinct motivational mechanisms (Pintrich, 2000). Consequently, these goals drive different learning behaviors and outcomes in dental training. Based on the theories mentioned above, we propose the first hypothesis:

*H1*: The four types of achievement goals influence the scholastic achievement of dental students to varying degrees.

As dentistry is a discipline that combines medical knowledge and skills, it is necessary to examine the influence of different types of achievement goals on dental students’ scholastic achievement. However, the underlying relationship between achievement goals and scholastic achievement remains unclear. Learning engagement represents a continuous and positive state of mind related to learning, which is positively linked to scholastic achievement. Learning engagement is a positive, fulfilling, and learning-related state of mind that is characterized by vigor, dedication, and absorption (Schaufeli et al., 2002a). High levels of learning engagement indicate that students spend more time and energy on their studies and are therefore more likely to achieve better scholastic outcomes. The results of empirical research also show that there is a positive relationship between learning engagement and students’ scholastic achievement (Assefa et al., 2023; Casuso-Holgado et al., 2013; Huang et al., 2023). Different types of achievement goals can produce different emotional and behavioral processes, which in turn affect students’ learning engagement. According to self-determination theory (Ryan and Deci, 2000), the quality of students’ motivation—whether it is self-determined (intrinsic) or controlled (extrinsic)—profoundly shapes their learning experiences. Achievement goals represent specific manifestations of these motivational states, thereby serving as primary drivers of students’ learning engagement. A previous study reported that achievement goals affect learning engagement (Yang et al., 2023; Zhong et al., 2023). Furthermore, another study demonstrated that performance orientation and mastery orientation have positive effects on learning engagement (Xie et al., 2022). These findings suggest that achievement goals are related to scholastic achievement through learning engagement. The Stimulus-Organism-Response (S-O-R) framework and learning engagement theory provide strong theoretical support for this mediating pathway (Astin, 1984; Fredricks et al., 2004). Motivation alone cannot directly produce academic outcomes; it must translate into active behavioral, emotional, and cognitive involvement (Kuh, 2009). Achievement goals act as internal motivational stimuli that guide how students allocate time and effort to their studies. High engagement directly facilitates knowledge retention, deeper clinical reasoning, and better examination performance (Christenson et al., 2012). Therefore, learning engagement functions as an essential behavioral mechanism linking internal goal orientations to observable academic success. Therefore, we propose the following second hypothesis:

*H2*: Learning engagement mediates the relationship between achievement goals and scholastic achievement.

At present, empirical evidence on the mediating role of learning engagement in the association between achievement goals and scholastic achievement remains limited, particularly in health-related educational contexts (Lei et al., 2024; Montenegro and Schmidt, 2023). This gap is particularly relevant in dental education, which requires the integration of theoretical knowledge and clinical skills, thereby requiring both high motivation and engagement. Therefore, understanding how different types of achievement goals are associated with dental students’ scholastic achievement through learning engagement is essential for improving educational strategies in this field.

Prior studies have identified specific demographic characteristics that may be associated with differences in students’ achievement goals, learning engagement, and scholastic achievement. These factors include sex, college, only-child status, family atmosphere, and grade level. Male and female students often exhibit different motivational patterns. Previous studies have indicated that male students tend to exhibit higher levels of approach goals in medical education (Rother et al., 2024). Regarding institutional factors, previous studies have shown that students in elite universities often face a highly competitive environment, which may increase their performance goals (Cecalupo et al., 2022). Therefore, participants from these two institutions were included in this study because Sun Yat-sen University (SYSU) is one of China’s elite universities, whereas Bengbu Medical University (BMU) represents a regional medical university. Family factors are taken into consideration. A good family atmosphere and only-child status can provide strong emotional support and more resources. Therefore, these factors are positively related to students’ learning engagement and scholastic achievement (Jia et al., 2021; Xiang et al., 2025). First- and second-year students mainly study basic medical sciences such as anatomy and histology. Third-and fourth-year students progress to clinical courses such as medicine, surgery, and endodontics. This change in coursework may significantly affect students’ learning behavior. For example, in the clinical years (third and fourth years), the learning environment changes substantially. Students begin to treat real patients and face clinical skill tests. This real-world setting greatly strengthens their professional identity. Consequently, they often develop stronger mastery-approach goals (Ten Cate et al., 2011).

In summary, we designed the study to address the following research questions: (1) Do these demographic characteristics of dental students result in differences in achievement goals, learning engagement, and scholastic achievement? (2) Does learning engagement mediate the relationship between achievement goals and scholastic achievement?

## 2 Theoretical framework

Taken together, although prior studies have indicated the links between achievement goals, engagement, and scholastic achievement, the specific pathways connecting these variables remain unclear. In particular, the extent to which learning engagement mediates the relationship between different achievement goals and scholastic achievement among dental students has not been systematically investigated. This study is grounded in the self-determination theory (Ryan and Deci, 2000), which states that mastery-approach goals are closely related to intrinsic motivation. Intrinsic motivation inherently promotes vigor, dedication, and absorption, which are the fundamental dimensions of learning engagement. Avoidance goals are often driven by controlled motivation and fear of failure, which may deplete cognitive resources and adversely affect learning engagement (Schaufeli et al., 2002b). According to the motivation-engagement framework (Skinner et al., 2008) and achievement goal theory (Elliot and McGregor, 2001), students’ motivational orientations affect their academic outcomes primarily through behavioral and cognitive engagement. Dental education requires students to acquire theoretical knowledge and clinical skills simultaneously. In this situation, active learning engagement is the critical link that translates motivation into academic success (Liu et al., 2025). High levels of engagement facilitate students’ investment of the sustained time and effort required to master clinical competencies. Therefore, our study proposes a theoretical framework in which learning engagement acts as a mediator of the relationship between achievement goals and scholastic achievement (Figure 1). By testing this framework, the study aims to clarify the psychological mechanisms underlying academic performance in dental students and address the existing gap in the literature.

**Figure 1:**
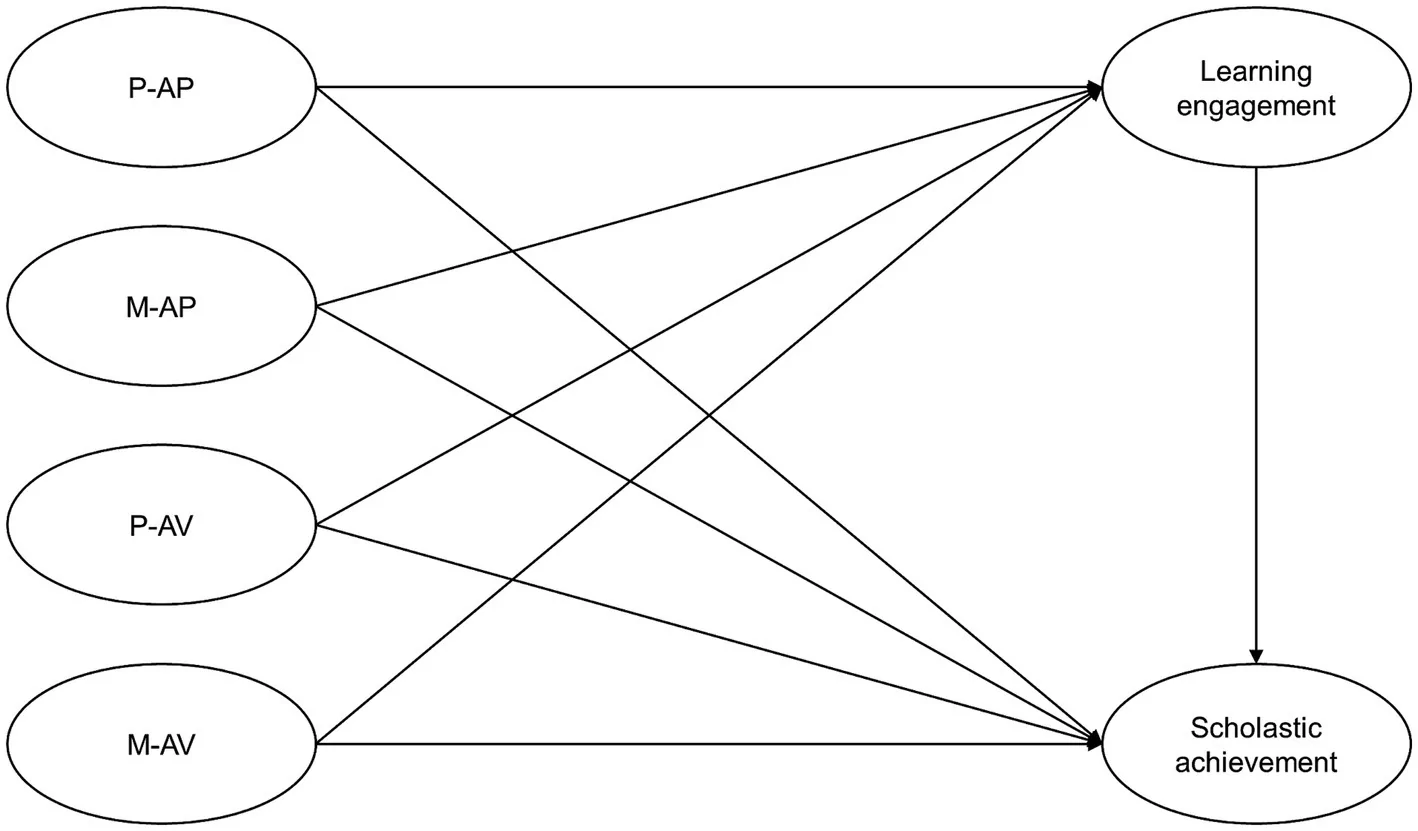
The relationship between achievement goals, scholastic achievement, and learning engagement. P-AP, performance-approach goals; M-AP, mastery-approach goals; P-AV, performance-avoidance goals; M-AV, mastery-avoidance goals.

## 3 Materials and methods

### 3.1 Procedure and participants

This quantitative cross-sectional study invited all first- to fourth-year dental students at Bengbu Medical University (BMU) and Sun Yat-sen University (SYSU). The participants consisted of male and female students majoring in dentistry who were 18 to 22 years of age. The online questionnaire was created using the Wenjuanxing platform and distributed via a web link. Between 15 July and 31 July 2023, the web link was distributed by the administrative staff of SYSU and BMU. Before completing the questionnaire, all participants were required to read and provide informed consent. The sample size of this study was determined based on established recommendations for structural equation modeling (SEM). Previous methodological studies suggest that a minimum sample size of 200 is required for reliable SEM estimation (Kline, 2015), with larger samples (e.g., 10 to 20 participants per estimated parameter, *n*:q ≈ 10–20:1) recommended for complex models (Wolf et al., 2013). Based on this principle, the required sample size for this study was calculated as 29 × 20 = 580. The final sample size (*n* = 679) in this study exceeds these recommended thresholds, indicating that the study had sufficient power to detect both direct and indirect effects within the proposed model. Bengbu Medical University is located in northern China, while Sun Yat-sen University is located in southern China. Sun Yat-sen University joined the “Project 985” and “Project 211,” which have greater educational resources and aim to train world-class medical professionals. Participation was voluntary and anonymous. The questionnaire consisted of four parts: demographic characteristics, achievement goal scale, learning engagement scale, and scholastic achievement scale. This study was approved by the Human Research Ethics Committee of the Hospital of Stomatology at Sun Yat-sen University (No. KQEC-2023-72-01).

### 3.2 Measures

#### 3.2.1 Demographic characteristics

Five demographic characteristics were assessed: gender, college, only-child status, family atmosphere, and grade. Students were divided into two categories: male and female. The students were recruited from two universities: Bengbu Medical University (BMU) and Sun Yat-sen University (SYSU). This study, covering dental students with different backgrounds, will better enhance the generalizability of the findings. Regarding only-child status, the students were divided into two categories based on whether they came from only-child families: “Yes” and “No.” The family atmosphere was divided into two categories: “Very good” and “Normal.” Based on participants’ subjective perceptions, family atmosphere was assessed as a self-reported binary variable. Previous studies have suggested that family structure may influence students’ motivation and academic outcomes (Dufur et al., 2013). Grade level was divided into two categories: “junior grade” and “senior grade.” “Junior grades” referred to first- and second-year students who mainly studied basic medical courses, such as biochemistry, anatomy, and immunology. “Senior grade” students referred to third- and fourth-year students who mainly studied clinical courses, such as internal medicine, surgery, endodontics, and prosthodontics. This was done to observe whether the differences in learning subjects between junior and senior grades would be associated with differences in the variables of interest.

#### 3.2.2 Achievement Goals Scale

Achievement goals were measured by the Chinese version of the Achievement Goals Scale and were shown to have good reliability and validity by previous studies (Liu et al., 2006; Mouratidis et al., 2018). It consisted of 4 types of achievement goals and included 29 items. The four types were performance-approach goals, mastery-approach goals, performance-avoidance goals, and mastery-avoidance goals. The performance-approach goals included 9 items that focused on being smarter and more capable than others. The mastery-approach goals included 9 items that were about understanding the task and improving proficiency. The performance-avoidance goals included 6 items and were about not appearing less intelligent or less competent than others. The mastery-avoidance goals included 5 items that revolved around avoiding mistakes or completing tasks they did not understand. Participants scored each item according to their actual situation, ranging from 1 (completely disagree) to 5 (completely agree), with higher scores indicating students’ higher levels of achievement goals. The Cronbach’s alpha (α) of the total scale was 0.92, and the four subscales were 0.88 for performance-approach goals, 0.87 for mastery-approach goals, 0.88 for performance-avoidance goals, and 0.82 for mastery-avoidance goals (Supplementary Table S1). The results of confirmatory factor analysis shown in Supplementary Table S2 indicated that the scale had good validity (*χ*^2^/df = 2.98, CFI = 0.91, TLI = 0.90, RMSEA = 0.05, and SRMR = 0.05).

#### 3.2.3 Learning Engagement Scale

Learning engagement was measured by the Chinese version of the College Student Learning Engagement Scale-student (UWES-S) scale (Fang et al., 2008), which was developed by Schaufeli et al. (2002a) and Schaufeli et al. (2002b). Lots of studies have used this scale to evaluate the learning engagement of students (Liu et al., 2023; Mouratidis et al., 2018; Wu et al., 2020). It consists of 3 dimensions and includes 17 items. The 3 dimensions are vigor, dedication, and absorption. The dimension of vigor includes 6 items that revolve around positive attitudes toward learning. The dimension of dedication includes 5 items that revolve around believing that learning is meaningful and being passionate about learning. The dimension of absorption includes 6 items that revolve around the concentration of energy during learning. Participants scored each item according to their actual situation, ranging from 1 (completely disagree) to 5 (completely agree), with higher scores indicating students’ higher engagement in learning. The Cronbach’s alpha (α) of the total scale was 0.95, and the three subscales were 0.90 for vigor, 0.86 for dedication, and 0.90 for absorption (Supplementary Table S1). The results of confirmatory factor analysis shown in Supplementary Table S2 indicated the scale had good validity (*χ*^2^/df = 2.77, CFI = 0.96, TLI = 0.956, RMSEA = 0.05, and SRMR = 0.04).

#### 3.2.4 Scholastic Achievement Scale

Scholastic achievement was measured by the Chinese version of the Scholastic Achievement Scale (Su, 2022). It consisted of 10 items that focused on the comprehensive assessment of test scores, after-school studies, sports scores, etc. Participants scored each item according to their actual situation, ranging from 1 (completely disagree) to 5 (completely agree), with higher scores indicating higher scholastic achievement. The Cronbach’s alpha (α) of the scale was 0.93 (Supplementary Table S1). The results of confirmatory factor analysis shown in Supplementary Table S2 indicated that the scale had good validity (*χ*^2^/df = 2.73, CFI = 0.98, TLI = 0.97, RMSEA = 0.05, and SRMR = 0.03).

### 3.3 Data analysis

SPSS 26.0 and Mplus 8.3 software were used to analyze the data. Harman’s single-factor test was used to check common method bias. Cronbach’s alpha (α) was used to evaluate the reliability of three scales. Confirmatory factor analysis was performed to test the validity of each scale. One-way analysis of variance was performed to evaluate the effects of college, gender, only-child or not, family atmosphere, and grade on the three scales. Pearson correlation was used to assess relationships between variables. A structural equation model (SEM) was used to examine the effects of achievement goals on scholastic achievement through learning engagement. Multigroup confirmatory factor analysis (MGCFA) was conducted to test the model invariance across the two universities. Measurement invariance was evaluated using recommended fit criteria (ΔCFI ≥ − 0.010 and ΔRMSEA ≤0.015; Chen, 2007; Cheung and Rensvold, 2002). Model fit indices below 3.0 for *χ*^2^/df, above 0.90 for CFI, TLI, below 0.08 for SRMR, and below 0.06 for RMSEA are recommended as best practice. The bootstrapping method with 1,000 resamples was used to verify mediating effects (Baron and Kenny, 1986; MacKinnon et al., 2007a).

## 4 Results

### 4.1 Test of common method bias

Harman’s single-factor test was used to check common method bias. The results demonstrated that 8 factors had eigenvalues greater than 1. The first factor explained 32.26% of the total variance, which is lower than the critical threshold of 40% (Podsakoff et al., 2003). Therefore, common method bias was not a significant concern in this study.

### 4.2 The overview of participants

A total of 792 questionnaires were collected, and 113 questionnaires were excluded due to respondents not majoring in dentistry, short answer times, or duplicate submissions. A total of 679 valid questionnaires were included, and the response rate was 85.73% (Figure 2). The sample consisted of 39.91% from Bengbu Medical University and 60.09% from Sun Yat-sen University. Approximately half of the participants were male (51.84%), in the junior grade (49.19%), and from an only-child family (42.12%). Most participants thought their family atmosphere was very good (65.83%). More sample characteristics are shown in Table 1.

**Figure 2:**
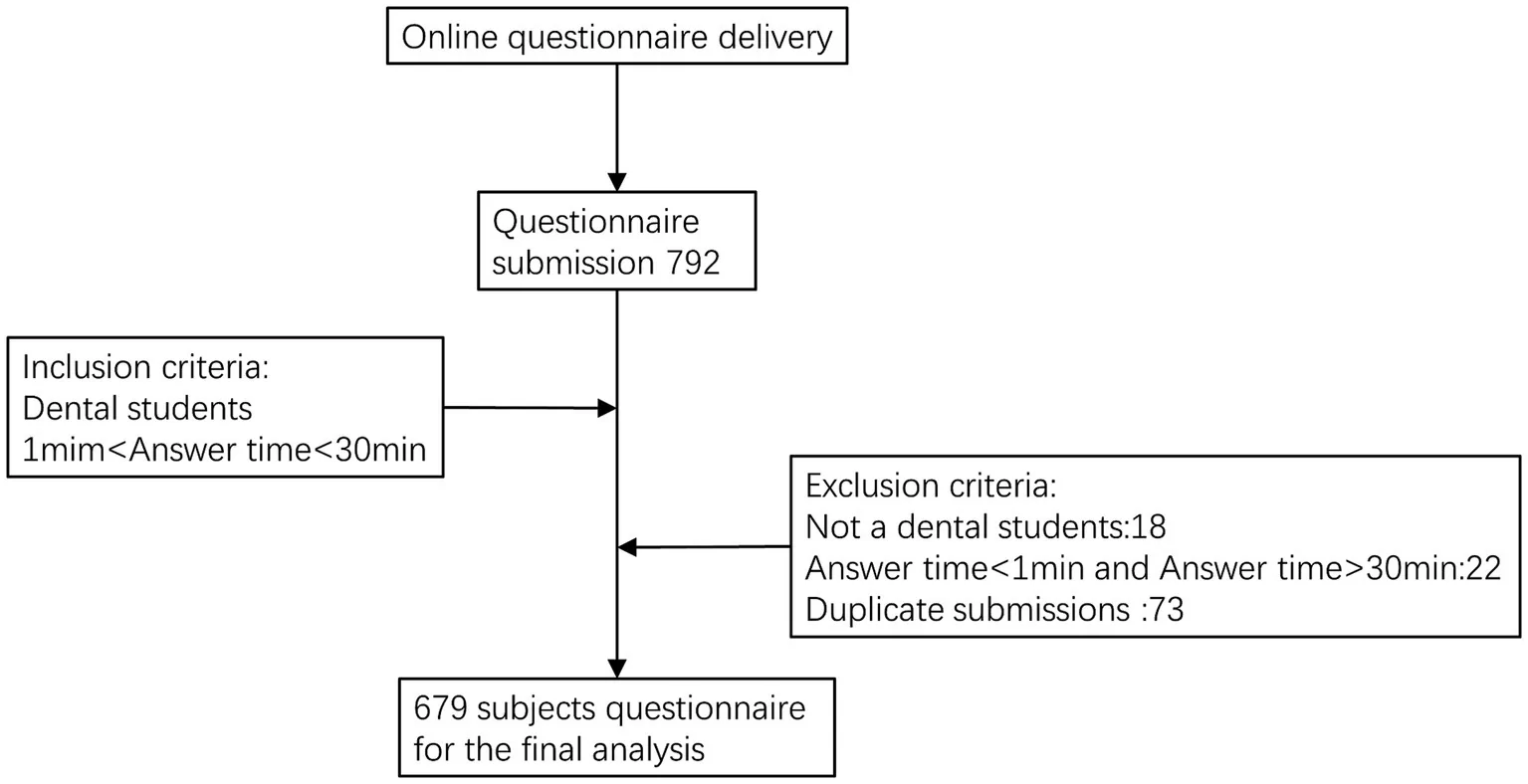
Flowchart of data acquisition.

**Table 1:** Statistical description and univariate analysis of variables in questionnaires.

Variable	Category	*N(%)*	Achievement goals *M* ± *SD*	Learning engagement *M* ± *SD*	Scholastic achievement *M* ± *SD*
College	SUSY^a^	408 (60.09)	3.17 ± 0.57	3.24 ± 0.64	3.41 ± 0.60
	BMU^b^	271 (39.91)	3.20 ± 0.63	3.45 ± 0.77	3.50 ± 0.74
	*p* (F)	/	0.52 (0.42)	**<0.001 (14.41)**	0.09 (2.84)
Gender	Male	327 (48.16)	3.22 ± 0.64	3.40 ± 0.74	3.49 ± 0.72
	Female	352 (51.84)	3.16 ± 0.55	3.25 ± 0.66	3.41 ± 0.60
	*p* (F)	/	0.19 (1.71)	**<0.01 (7.21)**	0.11 (2.58)
Only-child	Yes	286 (42.12)	3.18 ± 0.63	3.26 ± 0.72	3.45 ± 0.67
	No	393 (57.88)	3.18 ± 0.57	3.37 ± 0.69	3.45 ± 0.66
	*p* (F)	/	0.99 (0.01)	0.06 (3.58)	0.93 (0.01)
Family atmosphere	Normal	232 (34.17)	3.16 ± 0.57	3.19 ± 0.67	3.31 ± 0.62
	Very good	447 (65.83)	3.20 ± 0.61	3.39 ± 0.72	3.52 ± 0.67
	*p* (F)	/	0.35 (0.36)	**<0.001 (2.26)**	**<0.001 (2.53)**
Grade	Junior grade	334 (49.19)	3.21 ± 0.60	3.38 ± 0.75	3.51 ± 0.71
	Senior grade	345 (50.81)	3.16 ± 0.60	3.27 ± 0.66	3.39 ± 0.61
	*p* (F)	/	0.30 (0.55)	**<0.05 (3.42)**	**<0.05 (4.04)**
All	*/*	679 (100.00)	3.18 ± 0.60	3.32 ± 0.70	3.45 ± 0.66

^a^Sun Yat-sen University, ^b^Bengbu Medical University, df = 677. Bold values indicate statistically significant differences (*p* < 0.05).

### 4.3 The effect of general information

To evaluate the potential effects of demographic characteristics on the main variables, one-way analysis was performed. For achievement goals, the differences between groups were not statistically significant. For learning engagement, students from BMU (*p* < 0.001), male students (*p* < 0.01), students with a very good family atmosphere (*p* < 0.001), and junior grade students (*p* < 0.05) had higher learning engagement. For scholastic achievement, students with a very good family atmosphere (*p* < 0.001) and junior grade students (*p* < 0.05) had higher scholastic achievement. The above results and more details are shown in Table 1.

The results of general information on achievement goals are shown in Table 2. Students from BMU (*p* < 0.01), male students (*p* < 0.001), students with a very good family atmosphere (*p* < 0.001) and junior grade students (*p* < 0.05) had higher mastery-approach goals. Students with a very good family atmosphere (*p* < 0.05) had lower performance-avoidance goals. Students from BMU (*p* < 0.01) had lower mastery-avoidance goals.

**Table 2:** Statistical description and univariate analysis of achievement goals.

Variable	Category	*N(%)*	Performance-approach *M* **±** *SD*	Mastery-approach *M* **±** *SD*	Performance-avoidance *M* **±** *SD*	Mastery-avoidance *M* **±** *SD*
College	SUSY^a^	408 (60.09)	3.18 ± 0.79	3.44 ± 0.62	2.56 ± 0.89	3.41 ± 0.80
	BMU^b^	271 (39.91)	3.25 ± 0.81	3.59 ± 0.69	2.48 ± 0.99	3.28 ± 0.82
	*p* (F)		0.22 (1.45)	**<0.01 (8.35)**	0.31 (1.02)	**<0.05 (4.55)**
Gender	Male	327 (48.16)	3.24 ± 0.83	3.58 ± 0.68	2.50 ± 0.98	3.35 ± 0.84
	Female	352 (51.84)	3.17 ± 0.78	3.42 ± 0.61	2.55 ± 0.89	3.37 ± 0.77
	*p* (F)		0.25 (1.32)	**<0.001 (10.67)**	0.55 (0.37)	0.75 (0.01)
Only child or not	Yes	286 (42.12)	3.22 ± 0.87	3.49 ± 0.67	2.49 ± 0.95	3.40 ± 0.83
	No	393 (57.88)	3.21 ± 0.75	3.51 ± 0.63	2.55 ± 0.92	3.34 ± 0.79
	*p* (F)		0.87 (0.03)	0.71 (0.14)	0.44 (0.59)	0.34 (0.91)
Family atmosphere	Normal	232 (34.17)	3.16 ± 0.76	3.38 ± 0.61	2.65 ± 0.88	3.34 ± 0.75
	Very good	447 (65.83)	3.24 ± 0.82	3.56 ± 0.67	2.46 ± 0.95	3.37 ± 0.84
	*p* (F)		0.22 (0.99)	**<0.001 (2.44)**	**<0.05 (3.34)**	0.67 (1.83)
Grade	Junior grade	334 (49.19)	3.67 ± 0.90	3.56 ± 0.68	2.46 ± 0.94	3.39 ± 0.75
	Senior grade	345 (50.81)	3.56 ± 0.89	3.44 ± 0.62	2.59 ± 0.93	3.34 ± 0.66
	*p* (F)		0.13 (0.45)	**<0.05 (3.45)**	0.06 (0.01)	0.43 (0.42)
All	*/*	679 (100.00)	3.21 ± 0.80	3.50 ± 0.65	2.52 ± 0.93	3.36 ± 0.81

^a^Sun Yat-sen University, ^b^Bengbu Medical College University, df = 677. Bold values indicate statistically significant differences (*p* < 0.05).

### 4.4 The correlation analysis

The correlation analysis among the three scales is presented as a preliminary step to evaluate the feasibility of the mediation analysis in Table 3. The correlations between achievement goals and scholastic achievement were as follows: performance-approach goals (*r* = 0.48, *p* < 0.01), mastery-approach goals (*r* = 0.78, *p* < 0.01), performance-avoidance goals (*r* = 0.44, *p* > 0.05), and mastery-avoidance goals (*r* = 0.26, *p* < 0.01). The correlations between achievement goals and learning engagement were as follows: performance-approach goals (*r* = 0.41, *p* < 0.01), mastery-approach goals (*r* = 0.73, *p* < 0.01), performance-avoidance goals (*r* = 0.71, *p* > 0.05), and mastery-avoidance goals (*r* = 0.23, *p* < 0.01). There was a positive correlation between learning engagement and scholastic achievement (*r* = 0.77, *p* < 0.01).

**Table 3:** Correlations for achievement goals, learning engagement, and scholastic achievement.

Variable	1. Achievement goals	2. Performance-approach	3. Mastery-approach	4. Performance-avoidance	5. Mastery-avoidance	6. Learning engagement	7. Scholastic achievement
1. Achievement goals	1						
2. Performance-approach	0.87**	1					
3. Mastery-approach	0.72**	0.59**	1				
4. Performance-avoidance	0.68**	0.44**	0.13**	1			
5. Mastery-avoidance	0.76**	0.50**	0.41**	0.55**	1		
6. Learning engagement	0.49**	0.41**	0.73**	0.71	0.23**	1	
7. Scholastic achievement	0.51**	0.48**	0.70**	0.44	0.26**	0.77**	1

*p < 0.05, **p < 0.01, ***p < 0.001.

### 4.5 Model invariance between the two universities

Multigroup confirmatory factor analysis (MGCFA) was conducted to assess measurement invariance across the two universities (*N*_SYSU_ = 408, *N*_BMU_ = 271, Supplementary Table S3). The baseline configural model (M1) showed acceptable overall fit (*χ*^2^ = 7214.50, df = 2,912, CFI = 0.83, RMSEA = 0.07, SRMR = 0.07). Successively constraining factor loadings (metric model, M2: ΔCFI = −0.001, ΔRMSEA = 0.000) and item intercepts (scalar model, M3: ΔCFI = −0.005, ΔRMSEA = 0.000) yielded negligible fit changes, well within recommended cutoffs (ΔCFI = −0.010, ΔRMSEA = 0.015; Chen, 2007; Cheung and Rensvold, 2002). These results support full scalar invariance, confirming that the measurement instruments function equivalently across cohorts.

### 4.6 The effect of achievement goals on scholastic achievement

Structural equation modeling was used to analyze the relationships between achievement goals and scholastic achievement. In this model, the achievement goals were the exogenous latent variables (independent variables), and scholastic achievement was the endogenous latent variable (dependent variable). The fit indices of this model were as follows: *χ*^2^/df = 2.15, CFI = 0.91, TLI = 0.91, RMSEA = 0.05, and SRMR = 0.06. As shown in Figure 3, the effect of achievement goals on scholastic achievement was as follows: performance-approach goals (*β* = 0.15, *p* < 0.01), mastery-approach goals (*β* = 0.73, *p* < 0.001), mastery-avoidance goals (*β* = −0.10, *p* < 0.05), and performance-avoidance goals (*β* = −0.03, *p* > 0.05), respectively.

**Figure 3:**
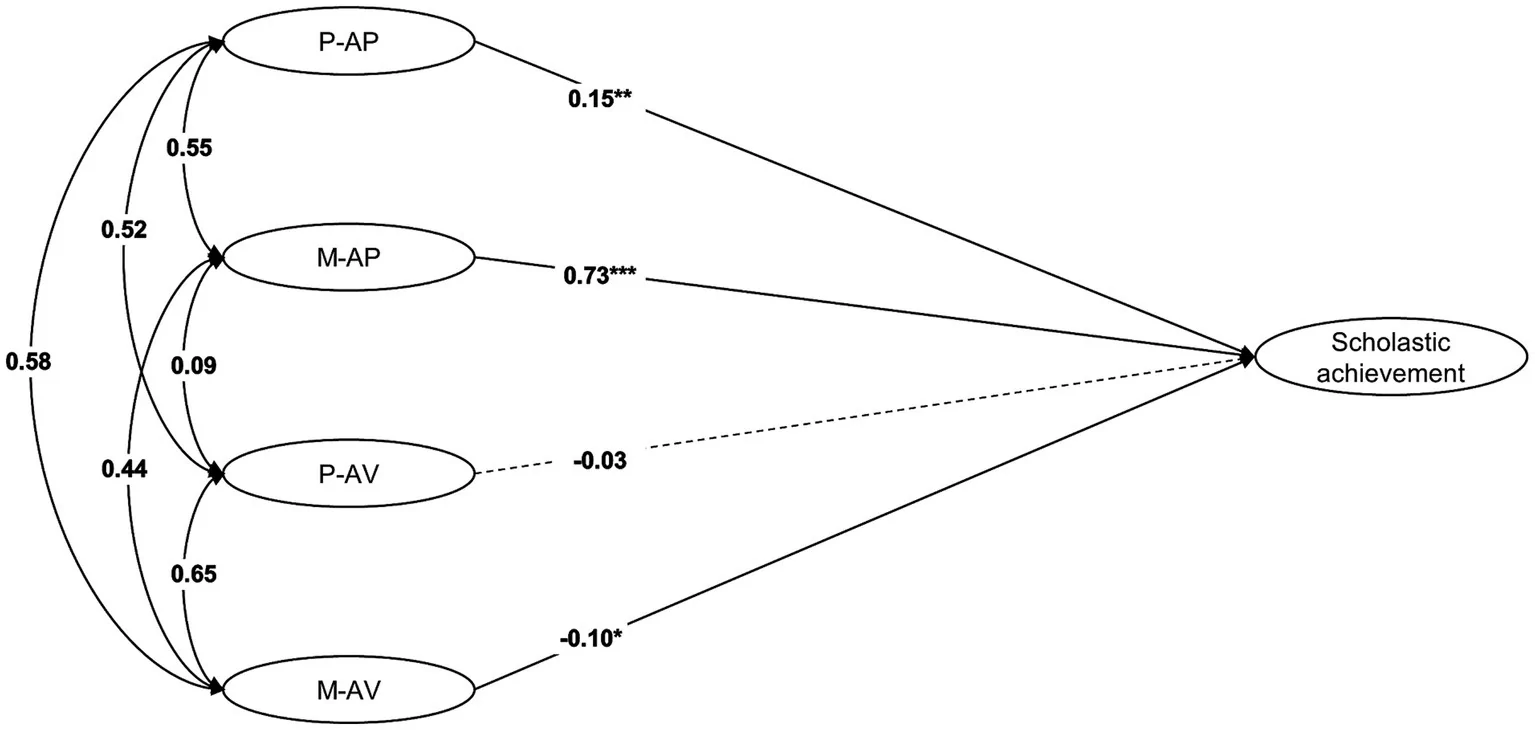
Structural equation model of the direct effect of achievement goals on scholastic achievement. This structural equation model shows the effects of the achievement goals scale on scholastic achievement. To make the model clear, the observed variables are not shown in the figure. The statistics are standardized path coefficients. Dotted lines represent non-significant paths; bold lines represent significant paths. P-AP, performance-approach goals; M-AP, mastery-approach goals; P-AV, performance-avoidance goals; M-AV, mastery-avoidance goals. *p < 0.05, **p < 0.01, ***p < 0.001.

### 4.7 Mediating effect of learning engagement

The bootstrapping method with 1,000 resamples was used to verify mediating effects (Baron and Kenny, 1986; MacKinnon et al., 2007a). Learning engagement was included as a mediator (mediating variable) in the model, and the results are shown in Figure 4. The fit indices of this model were as follows: *χ*^2^/df = 2.23, CFI = 0.91, TLI = 0.90, RMSEA = 0.04, and SRMR = 0.06. Achievement goals had different effects on learning engagement: performance-approach goals (*β* = −0.07, *p* > 0.05), mastery-approach goals (*β* = 0.94, *p* < 0.001), performance-avoidance goals (*β* = 0.13, *p* < 0.01), mastery-avoidance goals (*β* = −0.18, *p* < 0.001). Learning engagement affected scholastic achievement (*β* = 0.75, *p* < 0.001). As shown in Table 4, there were three significant mediating effects: learning engagement as a mediator of the relationship between mastery-approach goals and scholastic achievement (*p* < 0.01), learning engagement as a mediator in the relationship between performance-avoidance goals and scholastic achievement (*p* < 0.001), and learning engagement as a mediator effect in the relationship between mastery-avoidance goals and scholastic achievement (*p* < 0.01). Specifically, for mastery-approach goals and mastery-avoidance goals, the direct effects were not significant (mastery-approach goals: *β* = 0.03 and mastery-avoidance goals: *β* = 0.03). However, their indirect effects via learning engagement were both significant (mastery-approach goals: *β* = 0.70, 95% CI [0.549, 0.909]; mastery-avoidance goals: *β* = −0.13, 95% CI [−0.239, −0.047]). Because the indirect effects were significant while the direct effects were not, learning engagement acted as a full mediator in the relationships between mastery-approach goals or mastery-avoidance goals and scholastic achievement. In contrast, for performance-avoidance goals, both the direct effect (*β* = −0.13) and the indirect effect via learning engagement (*β* = 0.10, 95% CI [0.018, 0.193]) were significant, which indicated that learning engagement acted as a partial mediator. Finally, for performance-approach goals, the direct effect was significantly positive (*β* = 0.21), but the indirect effect through LE was not significant (*β* = −0.05, 95% CI [−0.132, 0.016], including zero). Thus, no mediating effect of learning engagement was established between performance-approach goals and scholastic achievement.

**Figure 4:**
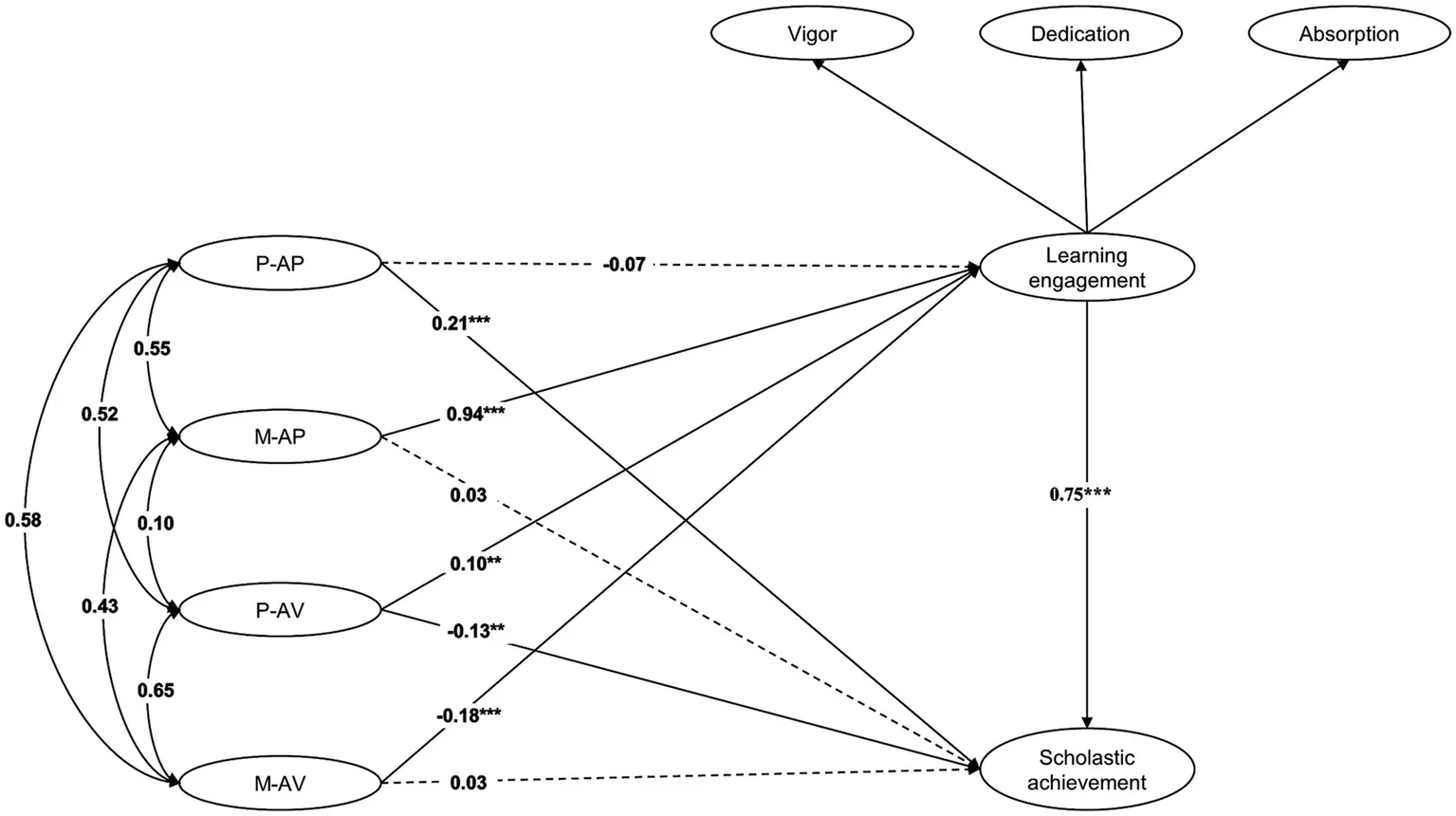
Structural equation model including learning engagement. This structural equation model shows the effects of the achievement goals scale on scholastic achievement, with learning engagement as a mediator. To make the model clear, the observed variables are not shown in the figure. The statistics are standardized path coefficients. Dotted lines represent non-significant paths; bold lines represent significant paths. P-AP, performance-approach goals; M-AP, mastery-approach goals; P-AV, performance-avoidance goals; M-AV, mastery-avoidance goals. *p < 0.05, **p < 0.01, ***p < 0.001.

**Table 4:** The mediating effect of learning engagement.

Mediation path	Direct effect	Indirect effect	95% BootCI
P-AP → LE → SA	0.21***	−0.05	[−0.132, 0.016]
M-AP → LE → SA	0.03	0.70***	[0.549, 0.909]
P-AV → LE → SA	−0.13*	0.10**	[0.018, 0.193]
M-AV → LE → SA	0.03	−0.13**	[−0.239, −0.047]

P-AP, Performance-approach; M-AP, Mastery-approach; P-AV, Performance-avoidance; M-AV, Mastery-avoidance; LE, Learning engagement; SA, Scholastic achievement. P-AP→LE→SA: Learning engagement as a mediator of the relationship between performance-approach goals and scholastic achievement, M-AP→LE→SA: Learning engagement as a mediator of the relationship between mastery-approach goals and scholastic achievement, P-AV→LE→SA: Learning engagement as a mediator of the relationship between performance-avoidance goals and scholastic achievement, M-AV→LE→SA: Learning engagement as a mediator of the relationship between mastery-avoidance goals and scholastic achievement. *p < 0.05, **p < 0.01, ***p < 0.001.

## 5 Discussion

The questionnaire efficiency was 85.73%, so the reliability of the questionnaire survey can be considered good. Among the 113 questionnaires excluded, the most common reason was duplicate submission (64.60%); the others were due to abnormal answer time (19.47%) and respondents not being dental students (15.93%).

The highest score on the achievement goals scale was for mastery-approach goals. It indicated that the achievement goals of dental students were mainly mastery goals, which meant that dental students paid more attention to improving their abilities and mastering the medical knowledge and skills. Dental students from SYSU showed lower scores on mastery-approach goals and higher scores on mastery-avoidance goals. This indicated that dental students at SYSU were more likely to avoid mistakes and to be more perfectionistic (Elliot and McGregor, 2001). Although traditional achievement goal theories suggest that elite academic environments should foster approach-driven motivation (Senko et al., 2011), our findings reflect the complex and stressful reality of elite dental education. This phenomenon may be explained by the fact that, compared with dental students from BMU, dental students from SYSU have higher admission scores and higher expectations for themselves, so when learning knowledge and mastering skills, they always have the psychological expectation that they can fully grasp them and avoid mistakes (Madigan et al., 2015).

Male students showed a higher level of mastery-approach goals than female students. This finding seems to contradict the well-established assumption that female students demonstrate higher mastery-approach goals (Meece et al., 2006). This divergence likely stems from the highly technical and spatial nature of dentistry. Previous studies have indicated that males generally report higher intrinsic interest and self-efficacy in complex mechanical tasks (Meece et al., 2006; Su et al., 2009). Since high self-efficacy strongly predicts approach-valenced motivation (Payne et al., 2007) male dental students’ greater confidence in mastering hands-on clinical procedures likely drives their higher mastery-approach goals. The learning of dental knowledge and skills is ultimately about mastering the ability to perform practical operations. Compared with female dental students, male dental students focus more on improving their knowledge and skills. Junior-grade dental students had a higher level of mastery-approach goals than senior-grade dental students. A meta-analysis of longitudinal studies showed that mastery-approach goals of college students declined with academic year (Scherrer et al., 2024). The specific causes of this phenomenon need to be investigated further.

Regarding the relationship between achievement goals and scholastic achievement, the results of the correlation analysis were consistent with those of the structural equation model. Performance-approach goals and mastery-approach goals were positively related to scholastic achievement, which is consistent with previous research (Frumos et al., 2023). These findings are also consistent with previous studies that mastery-approach goals are more strongly associated with deep learning strategies and sustained engagement, whereas performance-approach goals are often linked to outcome-oriented performance (Lei et al., 2024; Mouratidis et al., 2018). Medical students with performance-approach goals focus on achieving better grades in order to surpass others and prove their ability and superiority; therefore, students with performance-approach goals are likely to have good academic performance (Li et al., 2021). According to implicit theories of intelligence by Dweck (2013), a student with mastery goals has an ‘incremental’ mindset, and students with this mindset are good at gaining a sense of accomplishment in the face of challenges and through their own improvement and progress, even if the final result is failure. This mentality is also conducive to improved scholastic achievement. In our study, mastery-avoidance goals negatively affected scholastic achievement. However, studies have shown that even mastery-avoidance goals can positively affect scholastic achievement (Diaconu-Gherasim and Măirean, 2016; Frumos et al., 2023). This difference in results may be due to the different indicators used to measure scholastic achievement. In these two studies, only students’ grade points were used, whereas our questionnaire collected students’ comprehensive evaluation of themselves, including grades.

The results showed that mastery-approach goals, performance-avoidance goals, and mastery-avoidance goals are all mediated by learning engagement to affect dental students’ scholastic achievement. This finding can be further interpreted through established motivational and learning theories. From the perspective of Self-Determination Theory, different achievement goals reflect different types of motivation, which will influence how students engage in learning activities (Ryan and Deci, 2000). Mastery-approach goals are closely aligned with intrinsic motivation, which promotes deeper cognitive involvement and persistence and finally leads to enhanced learning engagement. In contrast, avoidance-oriented goals are often associated with controlled motivation and anxiety, which may either hinder or have inconsistent effects on engagement.

Moreover, the motivation–engagement framework suggests that motivation affects academic outcomes primarily through students’ behavioral and cognitive engagement (Skinner et al., 2008). This provides a theoretical explanation for the mediating role of learning engagement observed in this study. In the context of dental education, where both theoretical understanding and clinical skill acquisition are required, active engagement may serve as a critical pathway through which motivational orientations translate into scholastic achievement. Since the effect of mastery-approach goals and mastery-avoidance goals on scholastic achievement becomes statistically insignificant after the inclusion of learning engagement (Figures 3, 4), the mediating effect of learning engagement in the relationship between the two achievement goals (mastery-approach goals and mastery-avoidance goals) and scholastic achievement represents complete mediation (MacKinnon and Dwyer, 1993; MacKinnon et al., 2007b). This is one of the innovative findings of the study, which indicates that mastery-approach goals and mastery-avoidance goals affect the scholastic achievement of dental students mainly by regulating their learning engagement. In other words, a higher level of mastery-approach goals and a lower level of mastery-avoidance goals promote the learning engagement of dental students and then promote improved academic achievement. This pattern can also be interpreted through Expectancy-Value Theory (Eccles and Wigfield, 2002), which posits that students’ engagement is influenced by both their expectations of success and the perceived value of learning tasks. Students with mastery-approach goals are more likely to value learning itself and believe in their capacity to improve, which in turn promotes greater engagement and better academic outcomes. These findings suggest that greater attention should be paid to the training and guidance of students’ mastery goals in the training process of dental students, which is more conducive to their academic and professional development. Further research can provide a more detailed understanding by examining learning engagement in three sub-dimensions. Although performance-approach goals can affect scholastic achievement, this effect is not mediated by learning engagement. This result differs from some prior studies that reported a positive association between performance-approach goals and learning engagement (Xie et al., 2022). One possible explanation is that performance-approach goals may drive achievement through more surface-level or test-oriented strategies rather than sustained cognitive engagement. In highly competitive academic environments, such as dental education, students with performance-approach goals may prioritize short-term performance outcomes over deep learning processes, thereby weakening the mediating role of engagement. This finding indicates that different types of achievement goals may affect scholastic achievement through different paths. In addition to achievement goals, other factors in the motivation framework, or even factors based on other theoretical systems, may also influence scholastic achievement through the mediating effect of learning engagement.

## 6 Pedagogical implications

These findings offer practical insights for dental education. First, educators should actively foster dental students’ mastery-approach goals by encouraging students to focus on skill mastery rather than normative comparisons. Moreover, schools must prioritize dental students’ learning engagement. Given that learning engagement acts as a complete mediator between mastery goals and scholastic achievement, educators should adopt interactive and supportive teaching methods. By fostering positive achievement goals and building an engaging learning environment, educators can improve dental students’ scholastic achievement.

## 7 Limitations

Several limitations of this study should be acknowledged. First, the cross-sectional design limits the ability to draw causal inferences among achievement goals, learning engagement, and scholastic achievement. Future research should employ longitudinal tracking or experimental designs to confirm the causal relationships over time. Specifically, we tentatively hypothesize that an increase in mastery-approach goals at earlier academic stages will prospectively predict higher learning engagement and scholastic achievement in later academic stages. Second, this study was mainly limited to three variables: achievement goal orientation, learning engagement, and scholastic achievement. Incorporating more variables in the future would better reflect real-world complexities and enhance the reliability of the model. Key factors such as academic self-efficacy and test anxiety should be included. We tentatively hypothesize that academic self-efficacy may moderate the path between avoidance goals and learning engagement. Moreover, the assessment of family atmosphere relied on participants’ self-reported data. Utilizing standardized measurement scales would help to increase the credibility and validity of the research. Finally, all participants in this study were recruited from only two dental schools in China. Although these institutions represent different educational tiers (an elite university and a regional medical university), the overall sample may still limit the generalizability of our findings to students in other regions or health-related majors. Future studies should expand to multicenter cohorts across different cultural and geographical contexts.

## 8 Conclusion

This study explored the mediating role of learning engagement in the relationship between achievement goals and scholastic achievement. Mastery-approach goals demonstrated the strongest correlation with the scholastic achievement of dental students. Furthermore, learning engagement served as a complete mediator between mastery-approach goals and scholastic achievement. This finding contributes to a deeper understanding of students’ motivation, engagement, and scholastic achievement within the unique learning environment of dental education.

## Data Availability

The raw data supporting the conclusions of this article will be made available by the authors, without undue reservation.
