# Supplementary material for: The relationship between achievement goals and scholastic achievement in dental students: the mediating effect of learning engagement
Source: Front Psychol. 2026 Oct 8;17:1740774. doi: 10.3389/fpsyg.2026.1740774 (PMC13687435; doi:10.3389/fpsyg.2026.1740774)
Supplement: Supplementary file 1 [file Data_Sheet_1.pdf]

The Impact of Achievement Goal Orientation on Academic Performance: The Mediating Role of Learning Engagement

Dear Students,

Thank you for participating in this survey! This study aims to understand your demographic background, achievement goal orientations, and academic outcomes. Your responses will help us understand the potential factors influencing academic achievements. In accordance with the Statistics Law of the People's Republic of China, your personal information and responses will be kept strictly confidential and used solely for academic research.

Part I: Demographic & Background Information

1. **Your Grade Level (August 2022 – July 2023):**

A. Freshman (1st Year)

B. Sophomore (2nd Year)

C. Junior (3rd Year)

D. Senior (4th Year)

2. **Student Origin (Place of Origin / Domicile): ____________________**

3. **Gender:**

A. Male B. Female

4. **Are you an only child?**

A. Yes B. No

5. **Family Atmosphere / Environment:**

A. Poor B. Fair / Average C. Very Good

Part II: Achievement Goal Orientation Scale

Instructions: Please read each statement carefully and rate the extent to which each statement reflects your actual thoughts and feelings. Please mark a check (✓) in the corresponding box. (Only select one option per item)

Rating Scale: 1 = Never, 2 = Rarely, 3 = Sometimes, 4 = Often, 5 = Always.

No.	Statement	Never	Rarely	Sometimes	Often	Always
1	I like learning because it helps me expand my knowledge.					
2	When studying, my main concern is that others will think I am dumb.					
3	I feel very good if I am the only person in class who can answer the teacher's question.					
4	During exams, I often worry that I might repeat the same mistakes I made before.					
5	I always treat the mistakes I made as a key focus in my learning.					
6	In class, I always try to perform better than others.					
7	In class, my primary desire is to learn as much as possible.					
8	In my studies, I am most afraid that the teacher might think I am dumb and unable to learn.					
9	I always want to prove my competence through my actions.					
10	In order to thoroughly					

	understand what was taught in class, I pay special attention to reviewing after class.					
11	I often worry that I may not completely understand the content taught in class.					
12	I feel very pleased when others admire my academic performance.					
13	My desire is to do better than the other students in my class.					
14	I like learning things that can increase my knowledge.					
15	If my question to the teacher doesn't get noticed, I worry that the teacher will think I am stupid.					
16	I do not take the initiative to speak in class because I don't want others to think I am inadequate.					
17	I always feel a sense of pleasure while studying.					
18	I am very pleased when I can attract others' attention during class activities.					
19	In class, I actively raise my hand to speak in order to verify whether my ideas/answers are					

	correct.					
20	I often worry that there are things taught in class that I have not fully mastered.					
21	When encountering difficult problems, I rarely ask my classmates mainly because I fear they will laugh at me.					
22	I like looking for opportunities to acquire new knowledge.					
23	Before every exam, I always worry that there are materials I haven't reviewed yet.					
24	Being publicly praised by the teacher makes me feel that I am superior to others.					
25	I like challenging learning materials because I can learn new things from them.					
26	I always try to achieve the best grades to prove that I am better than others.					
27	During exams, I often worry that I might have misunderstood the instructions/questions.					
28	When given opportunities for open class discussion, I often stay silent for fear that my ideas are naive.					

29	The goal of my hard work in studying is to improve my rank in the class.					

*Note:*Performance-approach: Items 3, 6, 9, 12, 13, 18, 24, 26, 29;Mastery-approach: Items 1, 5, 7, 10, 14, 17, 19, 22, 25;Performance-avoidance: Items 2, 8, 15, 16, 21, 28;Mastery-avoidance: Items 4, 11, 20, 23, 27

Part III: Utrecht Work Engagement Scale for Students (UWES-S-17)

Instructions: Please indicate the degree to which you agree or disagree with the following statements regarding your studies.

Rating Scale: 1 = Strongly Disagree, 2 = Disagree, 3 = Neutral / Undecided, 4 = Agree, 5 = Strongly Agree.

No.	Statement	Strongly Disagree	Disagree	Neutral / Undecided	Agree	Strongly Agree.
1	When I get up in the morning, I feel like going to class / studying.					
2	When I'm studying, I feel like I am bursting with energy.					
3	Even when studying does not go smoothly, I do not give up and can persevere.					
4	I can continue studying for very long periods at a time without taking a break.					
5	When studying, even if I feel mentally fatigued, I can recover quickly.					
6	When studying, I feel strong and					

	vigorous.					
7	I find my studies challenging.					
8	My studies inspire me.					
9	I am enthusiastic about my studies.					
10	I am proud of my studies.					
11	I find my studies to be full of meaning and purpose.					
12	When I am studying, I forget everything else around me.					
13	Time flies when I am studying.					
14	When I am studying, my mind is only on my studies.					
15	It is difficult to detach myself from my studies.					
16	I am immersed in my studies.					
17	I feel happy when I am studying intensely.					

Note: Vigor: Items 1–6;Dedication: Items 7–11;Absorption: Items 12–17

Part IV: Academic Achievement / Academic Performance Scale

Instructions: Please evaluate your academic performance based on your actual situation.

Rating Scale: 1 = Very Low / Poor, 2 = Low / Below Average, 3 = Moderate / Average, 4 = High / Above Average, 5 = Very High / Excellent.

No.	Indicator / Item	Very Low / Poor	Low / Below Average	Moderate / Average	High / Above Average	Very High / Excellent
1	The extent to which I persevere in					

	overcoming difficulties to complete learning tasks.					
2	The extent to which I increase study time in order to complete tasks on time.					
3	My level of effort dedicated to studying.					
4	The overall quality of my learning and coursework.					
5	The degree to which I complete assignments within the specified time.					
6	The extent to which my completed assignments and coursework meet the teacher's requirements.					
7	The extent to which my learning results achieve my expected goals.					
8	The frequency/degree of proactively seeking challenging learning tasks.					
9	The frequency/degree of proactively resolving problems that arise in learning.					
10	Overall, my academic standing and ranking in the whole class.					

Closing Statement:

Thank you very much for your time and participation! The information you have provided is invaluable to our research.

成就目标定向对学习成绩的影响：学习投入的中介作用

亲爱的同学： 感谢你参与本次调查！本问卷主要了解你目前的基本信息、成就目标定向以及学业成果，你的回答将帮助我们了解生源地对于学业成果的影响和发现可能影响学业成果的因素。对你填答的信息，课题组将严格遵守《中华人民共和国统计法》予以保密。

一、 个人基本信息

1. 您 2022.8-2023.7 月所在年级: A.大一 B.大二 C.大三 D.大四

您的性别：A.男 B.女

2.您的生源地为:______

3. 您的性别：A.男 B.女

4. 您是否独生子女： A.是 B.否

5. 您的家庭氛围: A.较差 B.一般 C.非常好

二、 问卷调查题目

问卷一 成就目标定向问卷

指导语：请将你的实际情况和感受与下面的各种描述对照，并根据你的真实情况在每个项目后面合适的数字上打勾(√)。1——“从不”; 2——“偶尔”; 3——“有时”; 4—“经常”; 5——“总是”。序号请在方格中打作答，每道题目只勾选一个答案

序号	请在方格中打勾作答,每道题目只勾选一个答案	从不	偶尔	有时	经常	总是
1	我喜欢学习是因为它能让我增长知识					
2	学习时，我最担心的是别人认为我很笨					
3	如果班上只有我一个人能回答出老师的提问，我会感觉非常好					
4	考试时我经常担心自己再犯以前出过的错.					
5	我总是把出错的地方作为学习的重点					
6	在班上，我总是尽量争取比别人表现得更出色					
7	上课时，我最大的愿望是能学到尽可能多的东西					

8	学习中，我最怕老师说我笨，学不会					
9	我总是希望通过自己的行动证明我很能干					
10	为了透彻理解课上所学的知识，我特别注意课后复习					
11	我常常担心自己不能透彻地理解课上所学的内容					
12	别人羡慕我的学习成绩，我会非常高兴					
13	在班里，我的愿望是比别人做得好					
14	我喜欢学习能让我增长知识的东西					
15	如果自己问老师问题没有引起注意，我担心老师会认为我很笨					
16	课堂上我不主动发言是不想让别人觉得自己很差					
17	在学习时我总是有愉快的感觉					
18	在活动中能引起别人的注意，我会很高兴					
19	在课堂上，为了验证自己的想法(答案)是否正确，我总是积极举手发言					
20	我经常担心课上还有没学会的东西					
21	遇到难题，我很少问同学，主要是怕同学笑话					
22	我喜欢寻找获得新知识的机会					
23	每次考试之前，我总担心自己还有没复习到的东西					
24	老师若当众表扬我会使我觉得自己比别人强					
25	我喜欢具有挑战性的学习材料，因为我能够从中学到新东西					
26	我总是试图争取考出最好的成绩以证明自己比别人更优秀					
27	考试时，我常担心自己错误地理解了题目的					

	要求.					
28	上课自由发言时，我常担心自己的观点幼稚而不敢说话					
29	我努力学习的目的是提高自己在班上的排名					

注：成绩趋近：3 6 9 l2 13 18 24 26 29，掌握趋近：1 5 7 10 14 17 19 22 25 成绩回避：2 8 15 16 21 28。掌握回避：4 11 20 23 27

问卷二 学习投入问卷

指导语：请将你的实际情况和感受与下面的各种描述对照，并根据你的真实情况在每个项目后面合适的数字上打勾(√)。1——“完全不符合”; 2——“比较不符合”; 3——“不确定”; 4—“比较不符合”; 5——“完全不符合”。序号请在方格中打作答，每道题目只勾选一个答案

序号	请在方格中打勾作答,每道题目只勾选一个答案	完全不符合	比较不符合	不确定	比较不符合	完全不符合
1	早晨一起床，我就乐意去学习					
2	学习时，我感到精力充沛					
3	即使学习不顺利，我也毫不气馁，能够坚持不懈					
4	我能持续学习很长时间,中间不需要休息					
5	学习时，即使精神疲劳，我也能很快恢复					
6	学习时，我浑身有力而且干劲十足					
7	我发现学习富有挑战性					
8	学习激发我的灵感					
9	我对学习充满热情					
10	我因我的学习而感自豪					
11	我发现学习目的明确，而且很有意义					
12	学习时，我忘了周围的一切					

13	学习时，我感到时间过得很快					
14	学习时，我心里只想着学习					
15	我难以放下手中的学习					
16	我沉浸在学习中					
17	全身心投入学习时，我感到很快乐					

1-6 活力，7-11 奉献，12-17 专注

问卷三 学业成就问卷

指导语：请将你的实际情况和感受与下面的各种描述对照，并根据你的真实情况在每个项目后面合适的数字上打勾(√)。1——“很低”; 2——“较低”; 3——“中等”; 4—“较高”; 5——“很高”。序号请在方格中打作答，每道题目只勾选一个答案

序号	请在方格中打勾作答,每道题目只勾选一个答案	很低	较低	中等	较高	很高
1	我坚持克服困难以便完成学习任务的状况					
2	为了准时完成学习任务，我增加学习时间					
3	我在学习上的努力程度					
4	我的学习质量情况					
5	我在规定的时间内完成作业的情况					
6	我完成的学习任务符合老师要求的程度					
7	我的学习结果达到预期目标的情况					
8	我主动寻求挑战性学习任务的情况					
9	我主动解决学习中存在问题的情况					
10	总的来看，我的学习成绩排名在全班的位置					

结束语：

非常感谢您对此问卷调查工作的支持！您提供的信息对我们今后的工作非常有价值！
