# Supplementary material for: The relationship between achievement goals and scholastic achievement in dental students: the mediating effect of learning engagement
Source: Front Psychol. 2026 Oct 8;17:1740774. doi: 10.3389/fpsyg.2026.1740774 (PMC13687435; doi:10.3389/fpsyg.2026.1740774)

Supplementary Table 1. Reliability analysis of the scale.

Scale	Dimension	Cronbach's alpha	Item
Achievement goals	Performance-approach	0.88	9
	Mastery-approach	0.87	9
	Performance-avoidance	0.88	6
	Mastery-avoidance	0.82	5
	Total	0.92	29
Learning Engagement	Vitality	0.90	6
	Dedication	0.89	5
	Focus	0.90	6
	Total	0.95	17
Scholastic achievement	Total	0.93	10

Supplementary Table 2. The model fit indices of the confirmatory factor analysis .

	χ^2	df	χ^2/df	CFI	TLI	RSMEA	SRMR
Achievement goals	1070.10	359	2.98	0.91	0.90	0.05	0.05
Learning Engagement	316.23	114	2.77	0.96	0.96	0.05	0.04
Scholastic achievement	76.30	28	2.73	0.98	0.97	0.05	0.03

Supplementary Figure 1. SEM of Achievement Goals Scale

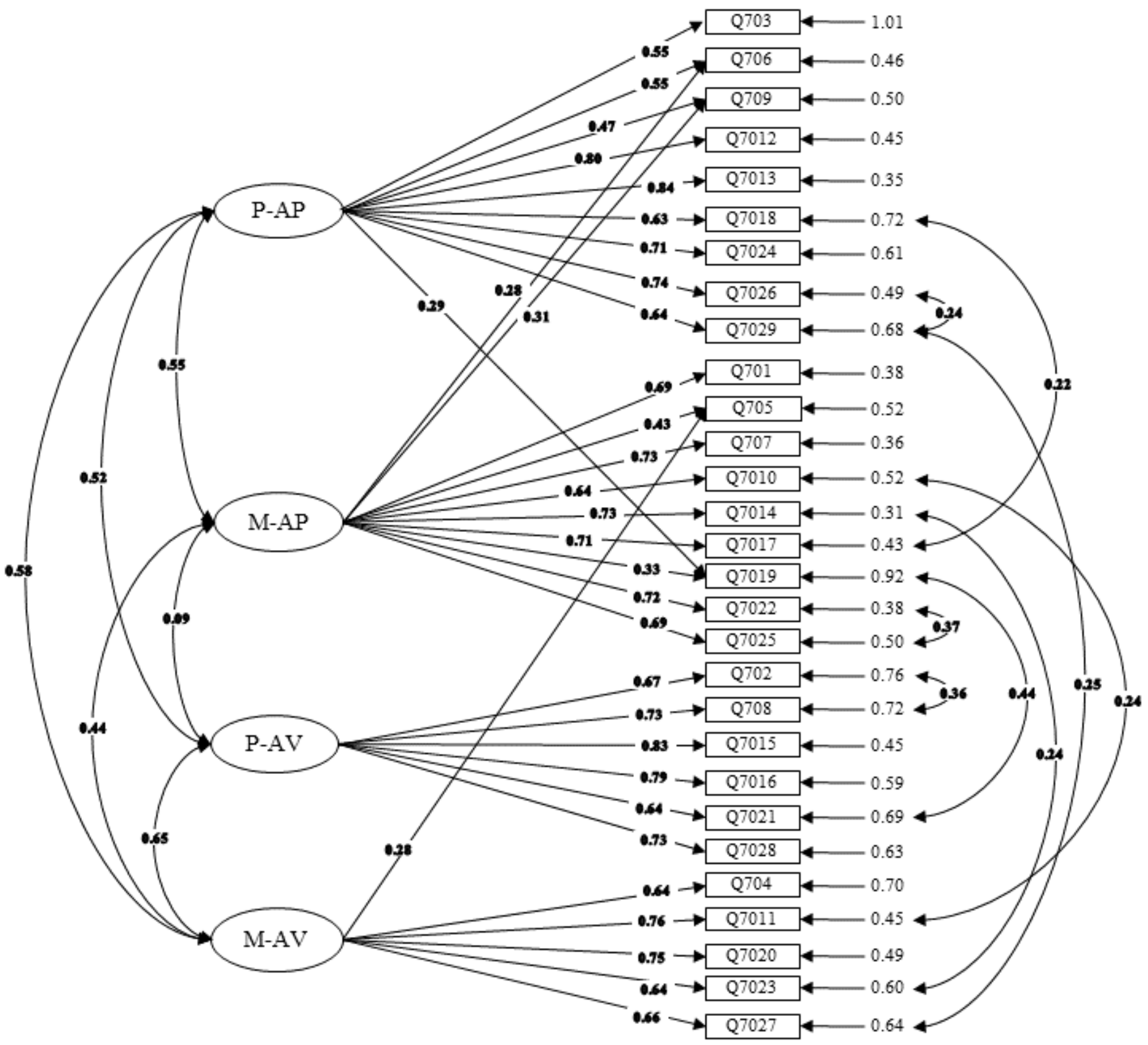

P-AP: Performance-approach, M-AP: Mastery-approach, P-AV: Performance-avoidance, M-AV: Mastery-avoidance.

Supplementary Figure 2. SEM of College Student Learning Engagement Scale-student scale

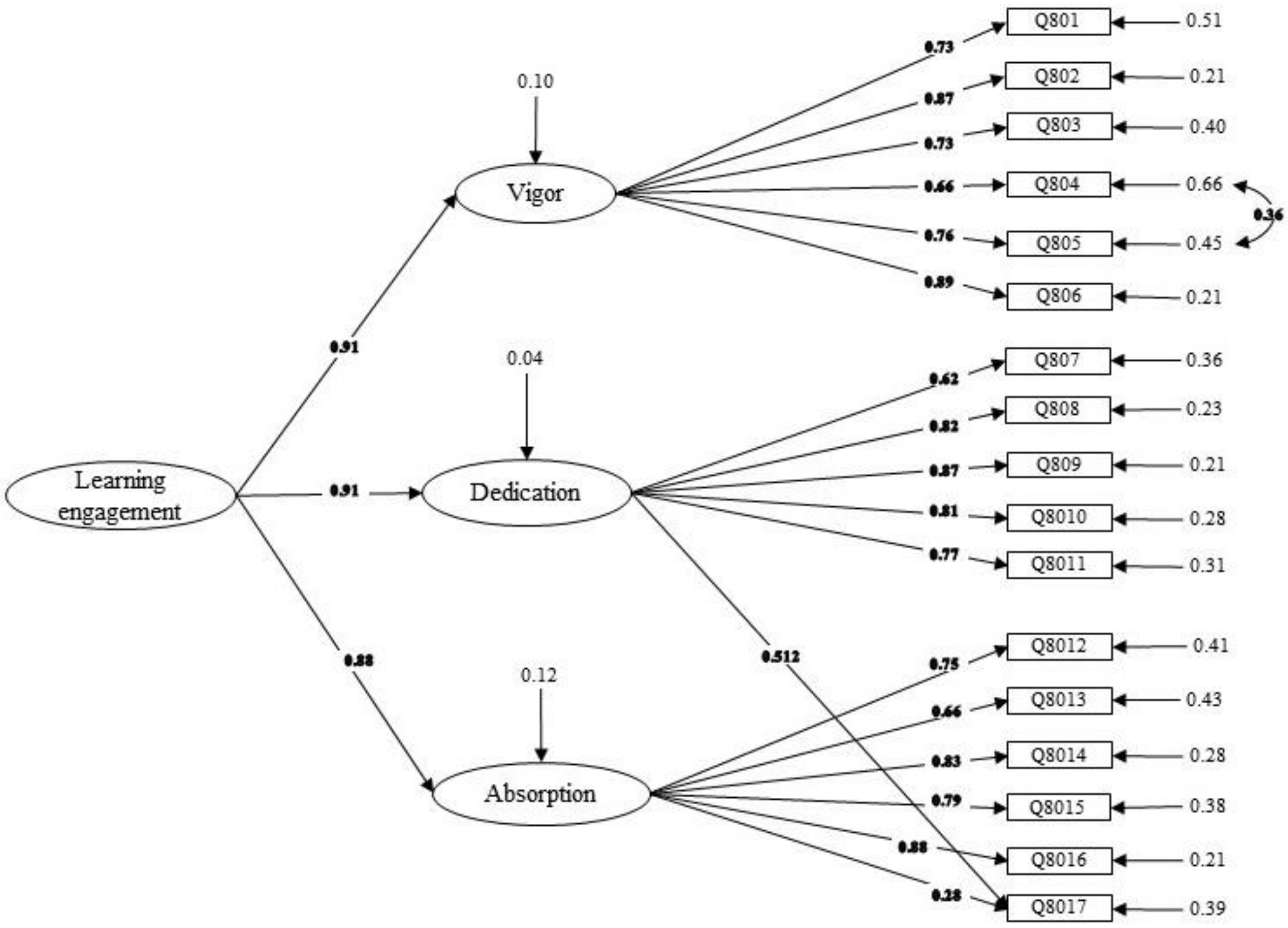

Supplementary Figure 3. SEM of Scholastic achievement Scale

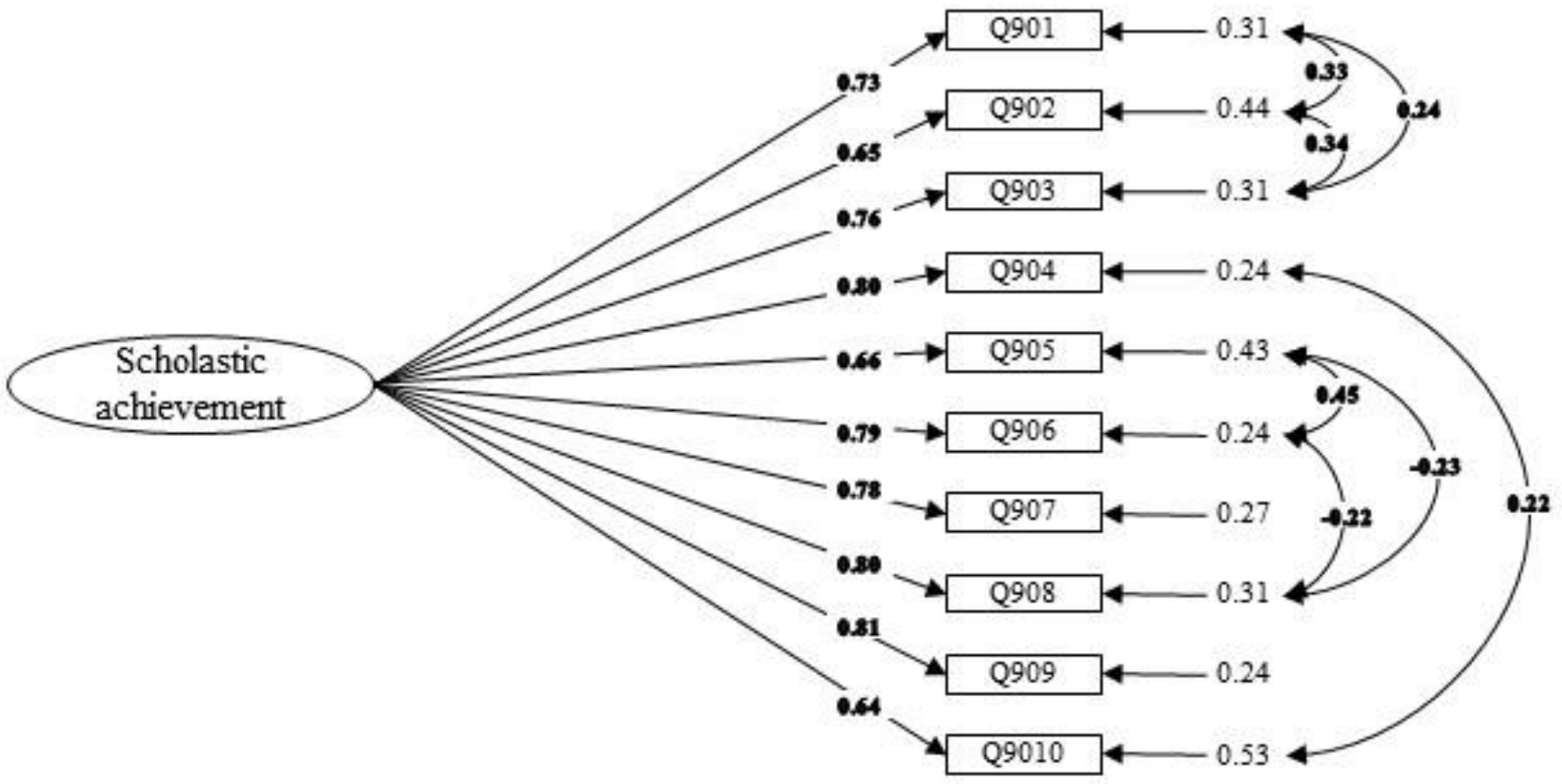

Supplement: Supplementary file 2 [file Supplementary_file_1.pdf]
